# Risk Assessment of Combined Exposure to Multiple Chemicals at the European Food Safety Authority: Principles, Guidance Documents, Applications and Future Challenges

**DOI:** 10.3390/toxins15010040

**Published:** 2023-01-04

**Authors:** Irene Cattaneo, Alexander D. Kalian, Matteo R. Di Nicola, Bruno Dujardin, Sara Levorato, Luc Mohimont, Alexis V. Nathanail, Edoardo Carnessechi, Maria Chiara Astuto, Jose V. Tarazona, George E. N. Kass, Antoine K. Djien Liem, Tobin Robinson, Paola Manini, Christer Hogstrand, Paul S. Price, Jean Lou C. M. Dorne

**Affiliations:** 1 Methodology and Scientific Support Unit, European Food Safety Authority, Via Carlo Magno 1A, 43216 Parma, Italy; 2 Department of Nutritional Sciences, Faculty of Life Sciences & Medicine, King’s College London, Franklin-Wilkins Building, London SE1 9NH, UK; 3 Unit of Dermatology, IRCCS San Raffaele Hospital, Via Olgettin 60, 20132 Milan, Italy; 4 Plant Health and Pesticide Residues Unit, European Food Safety Authority, Via Carlo Magno 1A, 43216 Parma, Italy; 5 iDATA Unit, European Food Safety Authority, Via Carlo Magno 1A, 43216 Parma, Italy; 6 Chief Scientist Office, European Food Safety Authority, Via Carlo Magno 1A, 43216 Parma, Italy; 7 Feed and Contaminants Unit, European Food Safety Authority, Via Carlo Magno 1A, 43216 Parma, Italy; 8 Retired United States Environmental Protection Agency (US EPA), 6408 Hoover Trail Road S.W., Cedar Rapids, IA 52404, USA

**Keywords:** risk assessment, human health, animal health, combined exposure, multiple chemicals, harmonised methodologies, component-based approach, assessment groups, future challenges

## Abstract

Human health and animal health risk assessment of combined exposure to multiple chemicals use the same steps as single-substance risk assessment, namely problem formulation, exposure assessment, hazard assessment and risk characterisation. The main unique feature of combined RA is the assessment of combined exposure, toxicity and risk. Recently, the Scientific Committee of the European Food Safety Authority (EFSA) published two relevant guidance documents. The first one “Harmonised methodologies for the human health, animal health and ecological risk assessment of combined exposure to multiple chemicals” provides principles and explores methodologies for all steps of risk assessment together with a reporting table. This guidance supports also the default assumption that dose addition is applied for combined toxicity of the chemicals unless evidence for response addition or interactions (antagonism or synergism) is available. The second guidance document provides an account of the scientific criteria to group chemicals in assessment groups using hazard-driven criteria and prioritisation methods, i.e., exposure-driven and risk-based approaches. This manuscript describes such principles, provides a brief description of EFSA’s guidance documents, examples of applications in the human health and animal health area and concludes with a discussion on future challenges in this field.

## 1. Introduction

Human and animal health risk assessment (RA) and ecological RA of the effects of combined exposure to multiple chemicals (“chemical mixtures”) is a challenging topic for academia, regulatory agencies, risk managers, industry and nongovernmental organisations. Assessing combined exposure requires the application of the classical steps of RA, namely problem formulation, exposure assessment, hazard assessment and risk characterisation [1]. There are, however, differences, including the integration of data on combined exposure and combined toxicity. In addition, two different approaches are used for risk characterisation, namely the whole mixture approach and the component-based approach; the selection of the approach depends on the level of characterisation of the mixture composition and the available exposure and toxicity data [2]. The whole mixture approach essentially considers the whole mixture of chemicals present in a product or environment media sample as a single chemical substance. As discussed below, the use of WMA is limited in human RA, animal RA, and ecological RA and is not discussed in detail in this review. Recently, the Scientific Committee of EFSA has published two guidance documents to harmonise methodologies for the risk assessment of combined exposure to multiple chemicals using CBAs, namely:

- “Harmonised methodologies for the human health, animal health and ecological RA of combined exposure to multiple chemicals” (MIXTOX guidance) [2].
- “Scientific criteria for grouping chemicals into assessment groups for human risk assessment of combined exposure to multiple chemicals” [3].

In addition, an online International Workshop on the RA of Combined Exposure to Multiple Chemicals was held in October 2021 and aimed to discuss these guidance documents and provide recommendations for future work in this field [1]. This review article presents a review of those documents and reflects a key-note presentation delivered by the corresponding author on 12 November 2021 during the Mycokey conference held in Bari, Italy. The RA principles for human and animal health in the area of multiple chemicals are introduced, key points from EFSA’s harmonised guidance documents are discussed, and examples are provided. This review concludes with a discussion on future challenges and recommendations to further improve methodologies in this complex area.

## 2. Principles and Harmonised Guidance Document for the Risk Assessment of Combined Exposure to Multiple Chemicals

### 2.1. Defining the Concept of “Mixtures” and the Three Types of Mixtures

The term “mixtures” is used to describe a range of combined exposures. The definition of a mixture has been proposed in Article 3 of the REACH regulation and Article 2 of CLP regulation and a “mixture” is defined as a mixture or solution composed of two or more substances [4,5]. However, in the context of food and feed safety for human health, animal health and ecological RA, exposures that may contribute to combined effects are not confined to chemicals present in the same medium, such as food, feed or an environmental media. Hence, EFSA has defined a mixture as “any combination of two or more chemicals that may contribute to effects on a receptor (human or environmental) regardless of source and spatial or temporal proximity” [2]. In addition, mixtures have been classified as intentional, unintentional or coincidental, based on [6].

Intentional mixtures are manufactured formulated products that are marketed with compositions tending to be consistent over time so that all exposed individuals can be assumed to be receiving proportionally similar doses of the mixtures’ components. The composition of an intentional mixture may be fully chemically defined or may be partially characterised. Examples of fully characterised mixtures are formulations such as a commercial pesticide, a food or feed additive, or a flavouring agent. Examples of products that are partially characterised include products that are produced by a controlled process, mixtures that are a combination of well-defined chemical mixtures to which an individual is exposed under a specific scenario, such as a group of individual plant protection products combined in a tank mix, or a group of commercial additives in a specific food. The Scientific Committee of EFSA notes that the term “chemically fully defined” does not mean that all chemical components have to be known. As with individual chemical substances, which, in practice, are never 100% pure, the acceptable impurities in a chemical mixture are usually defined in the specifications. Further, it is not possible to define a generic “cut-off” value, i.e., the minimum percentage of unidentified chemical substances that can be present in a mixture for it to be considered to be fully chemically defined, and below which it is considered partially or poorly defined, since this will be dependent on the nature of the mixture and of the possible impurities.

If a mixture is judged to be fully chemically defined, the preferred approach for mixture risk assessment is to use CBA, i.e., the risk is assessed based on exposure and effect data of its individual components. In contrast, RAs for less well-defined intentional mixtures require a whole mixture approach, in which the mixture is treated as a single entity.

Unintentional mixtures are sets of chemicals occurring in a single medium that occur as a result of industrial activities but which are not intentionally created. An example of such mixtures are the sets of pesticides in individual food items that result from the use of plant protection products on the crops that are ingredients of the food item. An example in ecological RA are the multiple chemicals released to the environment from a specific wastewater discharge during the production, transport, use or disposal of goods or environmental contamination or soil from petroleum wastes.

In contrast to intentional mixtures, the composition of unintentional mixtures is not fixed and may vary over time and space. For example, discharges from industrial activities vary from one discharge to another and the composition of a single discharge would vary over time. While such mixtures would also be considered less well defined, these mixtures cannot be evaluated using a whole mixture approach, since the composition of the mixtures is variable. As a result, CBAs are frequently used for unintentional mixtures. An exception to this pattern are whole mixture approaches used in ecological RA for effluents and water bodies (e.g., whole effluent toxicity testing [7]).

Coincidental mixtures are sets of doses that are received by a receptor (human or animal) from multiple sources and through multiple pathways. Examples of coincidental mixtures include combined doses of pesticides from an individual’s total diet and residential use of pesticides. Coincidental mixtures are not defined by the set of chemicals in any product, discrete food item or media. They are defined by the doses received by the receptor from the combined exposure and are a function of the number and nature of sources of exposure for the individual and the number and relative magnitude of the doses of the chemicals received from each source. These mixtures can vary greatly across individuals in an exposed population. The characterisations of these mixtures are performed by defining the ranges of doses for each chemical across the population and the correlations between the chemical-specific doses. Coincidental mixtures are typically assessed using probabilistic exposure models [8], duplicate dietary surveys [9,10] or biomonitoring studies [10]. Because there is no single discrete mixture, risks posed by coincidental mixtures cannot be assessed using whole mixture approaches.

### 2.2. Principles of Risk Assessment of Combined Exposure to Multiple Chemicals Using Component-Based Approaches

A common ground between single-substance and combined-substance RAs is the use of tiering principles. Tiering principles are applied in RA to allow for simple and conservative approaches at low tiers and more complex and refined approaches at higher tiers. Importantly, the appropriate application of tiering should reduce uncertainty of the risk assessment results, i.e., the higher the tier, the lower the uncertainty and the more closely the results resemble true exposures and impacts. In practice, the tiers can be qualified as low, intermediate or high or using numerical attributes (0, 1, 2, 3, etc.). A low-tier approach would typically be used to characterise risk in a data-poor situation and would require the use of conservative assumptions. A high tier would be used in a data-rich situation that allows the use of complex models including probabilistic approaches, including frequentist or Bayesian methods. It is important to note that the tier applied is not necessarily symmetrical between the RA steps, i.e., exposure assessment, hazard assessment and risk characterisation, because availability of data and regulatory requirements may vary across the steps. For example, for regulated products, the tier(s) to be applied can be predetermined by the available data, the problem formulation and/or the regulatory context [2,11,12].

Another common feature to RA of single and multiple chemicals is the use of mechanistic understanding to investigate toxicity through the mode of action (MoA) and adverse outcome pathway (AOP) frameworks.

**Figure 1:**
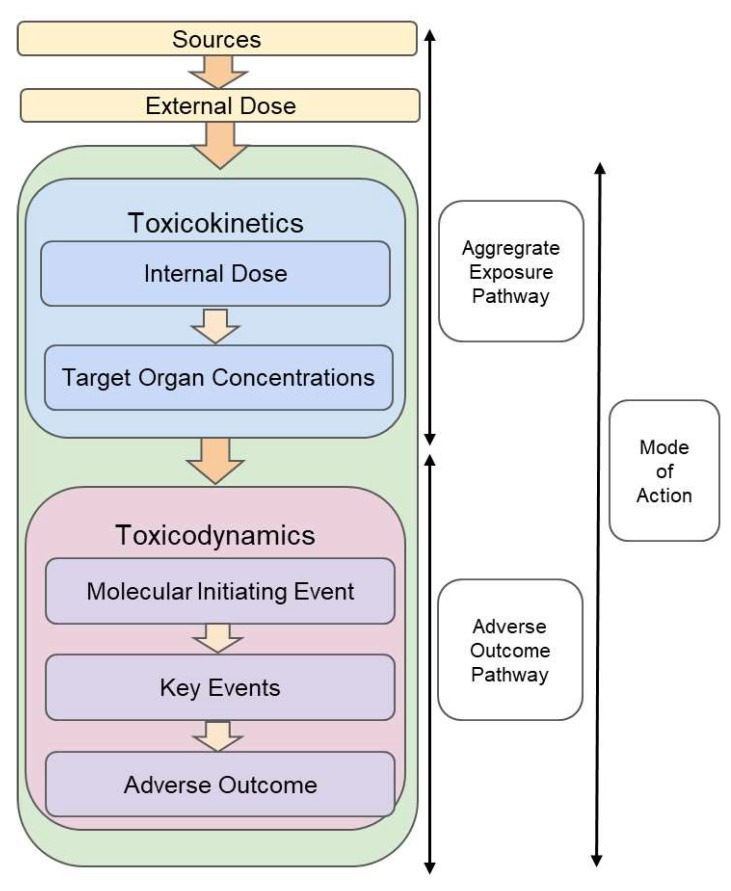
Mode of action, adverse outcome pathway, and aggregate exposure pathway (modified from [3]).

In essence, the MoA framework covers both the toxicokinetic (TK) dimension “what the body does to the chemical”, in other words, absorption, distribution, metabolism and excretion; and the toxicodynamic (TD) dimension “what the chemical does to the body”, in other words, toxicity [3]. In contrast, the AOP framework only covers the TD dimension. Recently, the aggregate exposure pathway (AEP) framework was proposed to cover the fate and transport, exposure and TK portions of the RA process. The AEP is designed to be an extension of the AOP and allows the integration of the exposure and TK dimension within a combined AEP–AOP framework. This joint framework accounts for interactions between chemicals that occur during the exposure, TK and TD steps of the RA [13,14]. Figure 1 highlights the differences between the MoA and AEP–AOP framework.

The CBA, which is the preferred and most often applied method in both human health and animal health areas for food and feed chemicals, applies both the tiering principles and the MoA and AEP–AOP frameworks, when data are available. In addition, applying the CBA requires a number of specific considerations, which differ from single-substance RA.

Grouping of chemicals into assessment groups and refinement of groupings is a process performed in the problem formulation (see Section 2.1 for details) after the identification and characterisation of the chemicals to be assessed. The multiple chemicals may be grouped using a number of shared properties, including regulatory criteria (e.g., pesticides, food and feed additives), exposure scenarios (e.g., same commercialised formulation), physicochemical properties, functional groups, chemical structures and shared toxicological properties (e.g., common mode of action, similar toxicokinetic properties, etc.). As data become more available, the grouping process can be refined using, for example, mechanistic data, such as data for MoA, AOPTK data or biologically based models (see Section 3 on hazard criteria for grouping) [2,3].

Dose addition is applied as the default assumption for the CBA so that all multiple chemicals behave as if they were dilutions of one another. If evidence on deviation from dose addition is available, such as interactions, other methods may apply (see below interactions).

Response addition implies the multiple chemicals have independent or dissimilar action, following the statistical concept of independent random events. Application of response addition has data requirements, i.e., toxicity data (e.g., mortality and target organ toxicity) to be expressed as a fraction (between 0 and 1). It is expressed as the percentage of individuals in a population or animal species in an ecosystem affected by the combined exposure or exceeding a reference point (e.g., no-observed adverse effect level (NOAEL)) or benchmark dose limit. Response addition has been qualified as a “misnomer” since responses are not actually added; instead, unaffected fractions of the population are multiplied.

Interactions refer to combined effects that differ from an explicit null model, i.e., dose and/or response addition, and are categorised as less than additive (antagonism, inhibition and masking) or greater than additive (synergism and potentiation).

A harmonised framework for the RA of combined exposure to multiple chemicals has been proposed in the EFSA MIXTOX guidance [2] with stepwise approaches starting from the problem formulation to the exposure assessment, hazard identification and characterisation (hazard assessment) and risk characterisation. The process is iterative and can be refined at any point. The harmonised guidance is described below for each of those steps [2].

### 2.3. Harmonised Guidance Document at the European Food Safety Authority: MIXTOX

#### 2.3.1. Problem Formulation

**Figure 2:**
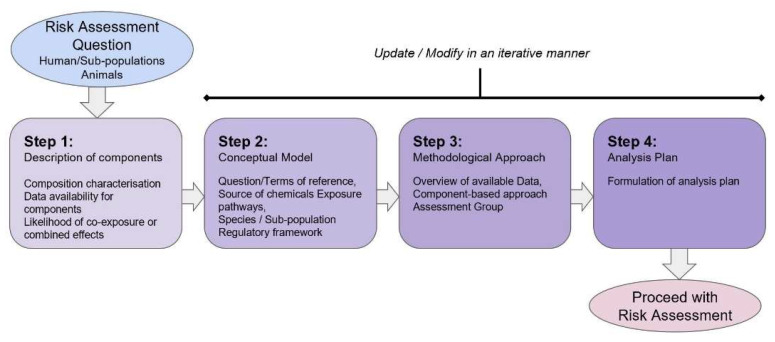
Stepwise approach for problem formulation of combined exposure to multiple chemicals using the component-based approach (modified from [2]).

Problem formulation provides the RA question considering the multiple compounds, species and/or subpopulations and food and feed items to be assessed and is a defined iterative process that is part of the dialogue between the originators of the request and the assessor. It aims to generate an analysis plan that describes what is the need, the extent and the methodology required for the RA to be performed [15,16]. In contrast to single chemicals, problem formulation for combined exposure to multiple chemicals can represent a complex phase and the stepwise approach is illustrated for MIXTOX in Figure 2.

The process initiates with the risk assessment question where the species under assessment are considered, namely humans and specific subpopulations (e.g., adults and children) or animals (e.g., farm or companion animals), together with the substances to be assessed. The stepwise approach for problem formulation is as follows:

- Step 1 deals with describing the components of the mixture and defines whether a combined exposure assessment is required according to the requestor’s question or the Terms of Reference. If so, the mixture composition needs to be characterised both qualitatively and quantitatively (e.g., if the mixture is intentional, is the composition poorly, partially or well-defined; if unintentional or coincidental, are the components and correlation between the components known?). At this stage, data availability is considered, including exposure in the species or population as well as hazard information, together with the likelihood of combined effects.
- Step 2 aims to develop a conceptual model to frame the RA itself, define data needs and suitable methods to be applied in subsequent assessment steps. The conceptual model is also the starting point for the assessment plan and the mathematical formulations of the models to be used during the exposure and the hazard assessment phases. At this stage, the identification of the origin or source of the chemical components of the mixture, the transfer pathway from the source to the target, the exposure pattern and the target populations and life stage exposed [17] can be included.
- Step 3 sets the method to be applied according to the exposure and toxicological data availability and can also be revisited depending on the outcome of the preliminary assessment.
- Step 4 provides an analysis plan to proceed with the RA process itself. The analysis plan may be modified, in the light of available new evidence, making it an iterative process.

#### 2.3.2. Exposure Assessment

Exposure assessment of combined exposure to multiple chemicals applies the same approaches as assessments of exposure to single chemicals (aggregate exposure) but can be more complex, since exposures to different chemicals can occur from separate sources and sequence of exposures can affect hazard. In essence, dietary exposure is assessed for each chemical in an assessment group, through integrating of occurrence data and consumption data. A common challenge to apply the CBA relates to differing quantity and quality of the data for different components, so that application of tiered approaches is required. A tier 0 approach would use default values for occurrence and consumption data, leading only to semi-quantitative point estimates of exposure. Tier 1 entails the use of somewhat more sophisticated data, such as modelled and experimental occurrence data, or consumption data. These can be generated from monitoring data such as food basket surveys in the food and feed safety area. Tier 1 assessments lead to deterministic exposure estimates. Tier 2 entails the use of more refined data, such as monitoring surveys for occurrence in individual food items or summary statistics for consumption data, which enables the determination of semi-probabilistic exposure estimates. Tier 3 are fully probabilistic assessments that can only be performed for the most data-rich RAs. Such assessments require individual co-occurrence data alongside individual consumption data. Tier 3 assessments produce more accurate information on the variation of exposure across individuals and the relative contributions of individual chemicals.

**Figure 3:**
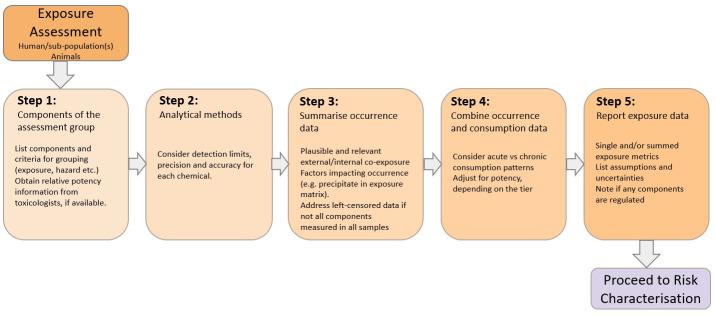
Stepwise approach for exposure assessment of combined exposure to multiple chemicals using the component-based approach (modified from [2]).

The stepwise approach for exposure assessment of multiple chemicals in the MIXTOX guidance (Figure 3) is as follows:

- Step 1: a list of the components within the assessment group is produced according to the grouping criteria (e.g., exposure, hazard, etc.) discussed in Section 3. Toxicologists are consulted to retrieve information on the relative potencies of individual chemicals and for defining the time frame when co-occurrence of exposures are relevant for the RA. For chronic and sub-chronic exposures, the co-occurrence timeframe for combined toxicity elicitation may vary depending on the kinetic profiles of the chemicals [8,18].
- Step 2: chemical occurrence data are collected and assembled, taking into account the plausibility of co-occurrence of individual components. When occurrence data specific to the target population are not available, data gaps can be filled from the available datasets on other populations that define the ratios and correlations between components. Occurrence data for each chemical need to originate from monitoring studies that use accurate and precise analytical methods. The data should, for example, note when concentration data are below the limit of detection or limit of quantification, as this could lead to left-censored data distributions that require specific considerations and corrections.
- Step 3: occurrence and consumption data are combined to estimate exposure through the use of appropriate tools depending on data availability and the selected methodology for risk characterisation [11,12]. Step 3 also foresees the calculation of potency-adjusted exposures starting from the toxicological advice provided in Step 1.
- Step 4: a summary report of exposure data with a comprehensive list of assumptions and uncertainties is produced. Specific exposure assessments may be performed for individual chemicals covered by an existing risk assessment or a defined legal framework.

#### 2.3.3. Hazard Identification and Characterisation

Hazard assessment (hazard identification and characterisation) for HRA and ARA of combined exposure to multiple chemicals using CBAs aims to derive quantitative hazard metrics, as reference points or reference values, for each chemical in the assessment group, with dose addition as the default assumption. The choice of the tier is driven by the purpose of the assessment and the data available, as described in the WHO, OECD and EFSA’s harmonised frameworks and is performed following data collection and evaluation. As hazard data are identified, the chemicals to be included in the various assessment groups maybe modified [2,3,11,12].

Reference points, such as NOAELs or benchmark dose limits, may be derived from in silico, read-across, in vitro and/or in vivo studies and observations in the population of interest and are used both for humans and animal species. Reference values are often expressed as health-based guidance values, such as acceptable or tolerable daily intake for regulated chemicals and contaminants, respectively, and are mostly used for HRA.

Data for different chemicals in the assessment group are often variable and incomplete. For tier 0, when reference points based on in vitro and/or in vivo studies are not available (data poor situations), data gaps can be filled using read-across for similar chemicals and in silico models to predict toxicity, such as quantitative structure activity relationship (QSAR) models or expert judgement through a structured expert elicitation. An example of this approach is the hazard assessment of ergot alkaloids (a group of structurally related contaminants [19]).

For tier 1, reference points become available, including NOAELs, BMDLs or a defined level of the common critical effect. At tier 2 and tier 3, more data are available and provide a greater understanding of toxicity from a mechanistic perspective. Such data provide the option to refine assessment groups as well.

Refinement of assessment groups can use a range of approaches, including weight of evidence (WoE) approaches, dosimetry or mechanistic data, when more hazard data are available [2,3,15].

WoE approaches can apply scoring systems to assess whether molecules should be grouped into the same assessment groups using data for known relevant properties and assigned relative weights that rank their significances. Dosimetry data may be used considering dose dependency for toxicity on target organs and sensitivity of different endpoints. Mechanistic data using in vitro assays or omics technologies may be used in order to refine according to common MoAs or AOPs for the same species.

Examples of refinement of assessment groups at tier 2 can involve the use of an index chemical, with a high-quality toxicological database allowing the calculation of relative potency factors for other components through dividing each reference point with that from the index chemical. In the mycotoxin field, relative potency factors have been applied for zearalenone and its modified forms [20]. Relative potency factors can also be used to estimate potency related exposure, such as toxic equivalent factors, as a type of relative potency factors expressed as equivalents of the index chemical. This approach is well known for dioxins and marine biotoxins, including okadaic acid and analogues [21,22]. Examples for tier 3 include the characterisation of MoA and AOPs in animals or humans based on in vivo, in vitro mechanistic information or epidemiological data and toxicokinetic studies, which then allow grouping to be refined and reference points to be derived based on internal dose using probabilistic approaches with biologically based models, as well as chemical-specific adjustment factors. These include physiologically based kinetic models [23,24,25].

Response addition is rarely used in the human health and animal health areas, since the reference points reflect a response level below the detection limit and experimental NOAELs often represent only a 1–10% response level, remaining undetected due to methodological constraints. Hence, applying response addition requires evidence of independent MoA between the individual and the multiple chemicals. In ecological RA, response addition is applied more often using the percentage of individuals in a population or species in an ecosystem showing a predefined effect (e.g., mortality, immobility or cancer) or exceeds a reference point or reference value (see the discussion on risk characterisation below).

Interactions can be dealt with at the hazard assessment, which is usually most appropriate for assessing the nature of the interactions and is dealt with subsequently in the risk characterisation step. Interactions can be of a TK or TD nature.

TK interactions have been shown to cause both antagonism and synergy through effects on the absorption, metabolism or transport of target chemicals. The specific consequence of these interactions will depend on whether the toxic moiety is the parent chemical or a metabolite. The magnitude of the TK interaction can be quantified using in vivo or in vitro TK parameters as the dose-dependent ratio between the TK parameters for the single chemical and the multiple chemicals. Examples include ratios of in vivo clearance for chronic exposure or TK models based on in vitro data to refine changes in internal exposure with constants of inhibition [26,27].

For TD interaction, the basis is of a mechanistic nature, i.e., interaction at the MoA or AOP triggered by each component. These differ from additivity and can be translated on the dose–response relationship of the individual components taking into account variations according to dose levels, the route(s), timing, duration of exposure and biological target(s) [28]. The magnitude and direction of the deviation defines whether there is synergism or antagonism and is quantified on the dose response for each chemical and the dose–response of the multiple chemicals through a model deviation ratio [29].

For either the TK or TD dimension, the magnitude of interaction can then be used to derive uncertainty factors to cover relevant percentiles of the species or population under assessment and, depending on the protection goals, can be taken into account in the risk characterisation. Recent examples include (a) quantification of changes in TK parameters for CYP3A4 substrates after grapefruit juice (CYP3A4 inhibition) and St John’s wort exposure (CYP3A4 induction) in humans and derivation of UFs [30], (b) in vivo dose–response modelling of the synergistic interaction between melamine and cyanuric acid (7- and 28-day studies) resulting in several fold increases in the nephrotoxicity of the melamine–cyanuric acid complex compared to that for the individual compounds [31,32].

Methods for risk characterisation of interactions are discussed in the next section.

**Figure 4:**
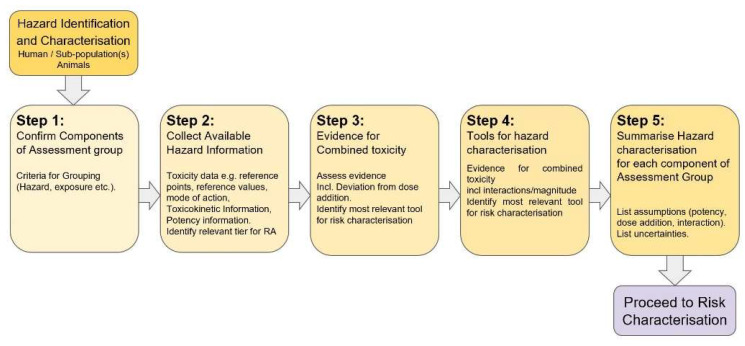
Stepwise approach for hazard identification and hazard characterisation of combined exposure to multiple chemicals using the component-based approach (modified from [2]).

The stepwise approach for hazard assessment (Figure 4) is as follows:

- Step 1: risk assessors have the opportunity to confirm or refine the initial grouping of chemicals performed at the problem formulation stage. If needed, a refinement can be made using WoE approaches, dosimetry (TK) or mechanistic data (i.e., MoA and AOP) [15,27,33].
- Step 2: the relevant entry tier [11,12] for the assessment is decided based on the purpose of the assessment and the available data. Hazard information is collected for each individual chemical and may include toxicity data, reference points, reference values, mechanistic data, toxicokinetic information and relative potency information. In case of data-poor situations, a list of possibilities to fill data gaps is identified.
- Step 3 assesses the evidence for independent action between individual chemicals of the assessment groups and the potential for interactions [18]. Within step 3, the most appropriate approach for risk characterisation is defined.
- In Step 4, for each individual component of the assessment group, reference point and uncertainty factors are derived to obtain appropriate reference values through the relevant tier. Such reference values can be used for individual components of the whole group (equivalents of an index chemical).
- Step 5 summarises the hazard metrics for individual components and lists assumptions and uncertainties.

#### 2.3.4. Risk Characterisation

In essence, the risk characterisation step aims to generate a ratio of combined exposure to the quantitative metric for combined toxicity for human or a defined animal species. If this comparison indicates that there is no safety concern, the assessment can be concluded. Alternatively, it indicates a signal to proceed to a higher tier, with the possible need for additional data, or an indication of a risk that is transferred to the risk management step [16,34]. This step requires careful interpretation and communication, particularly if the data used vary in quality, quantity or relevance. Uncertainties are identified in each stage of the framework and an overall uncertainty analysis is integrated in the risk characterisation [35].

For the CBA, tiering is also applied to accommodate the context of the assessment, data availability, time and resources. As tiers progress, more data-rich situations are considered and, hence, different risk characterisation methodologies may be used. The methodologies typically progress from default and conservative approaches to more quantitative and probabilistic approaches, with increasing consideration of internal dose using either experimental toxicokinetic data or toxicokinetic models [2].

At tier 0, the hazard index method is typically applied. Hazard index is calculated as the sum of component hazard quotients for a given assessment group, with the hazard quotient of each chemical defined as the ratio between exposure and reference values. If no reference values are available for a given chemical, the reference value that represents the most potent chemical in the assessment group may be used instead as a conservative estimate. The hazard index approach is regarded as a comparatively simpler and more time-efficient estimation method, particularly for data-poor scenarios where a conservative estimate may be required.

At tier 1, hazard index may still be applied; however, more data-rich situations may enable for the target organ toxicity to be used as part of a refined hazard index approach, which considers that adverse effects and target organs may vary between different components and, hence, commands endpoint-specific HI calculations. The reference point index may alternatively also be applied, which sums exposures of components with respect to reference points, with application of a single group assessment factor. The reference point index is inversely related to the combined margin of exposure as the reciprocal summation of margin of exposure values in each assessment group.

At tier 2, the reference point index approach may be used to calculate potency-adjusted exposure, for comparison to the index chemical reference point in order to derive a margin of exposure. If toxic equivalent factors are available, a reference value may be calculated for the most extensively studied (and typically most potent) component for use as a group reference value (expressed as toxic equivalents), leading to risk characterisation being a comparison of exposure and the group reference value.

For Tier 3, missing risk metrics are quantitative and probabilistic, while increasingly taking into consideration internal dose using either TK data or physiologically based TK or TK-TD modelling. Methods such as the internal dose HI correct exposure for internal dose, taking into account TK parameters, such as absorption or body burden. The internal dose correction can be applied to all methods described above, i.e., hazard index, reference point index, point of departure index and combined margin of exposure [34,36,37,38]. The application of probabilistic methods, such as probabilistic harmonic sum of margins of exposure derived from physiologically based TK-TD models and probabilistic exposure estimates for the assessment group components, constitute the most refined approach and have been applied using the probability of critical exposure from the distribution of individual margins of exposure model for the human health area [39,40]. However, as these methods require full TK and dose–response data for each chemical substance in the assessment group, they are rarely used in combined risk assessment [18,38,41,42,43].

At all tiers, the maximum cumulative ratio may be used and represents a metric of the degree in which a single chemical dominates mixture risks. The measure can be used to prioritise chemicals by identifying the individual chemicals contributing significantly to the toxicity in an assessment group. The maximum cumulative ratio represents the ratio of combined toxicity to the highest toxicity from a single chemical of the assessment group, with the minimum maximum cumulative ratio value being 1 and the maximum value being that of the number of chemicals in the mixture [44]. The maximum cumulative ratio is also a measure of the difference between risks predicted using dose addition models and response addition models [45].

Response addition may be considered if chemicals are deemed as likely to act via independent mechanisms, are deemed as unlikely to interact (either before or after the MoA) and also have response point data (as well as typically the complete dose–response data) available for at least two chemicals in the mixture. Response addition is mostly applied in the ERA area.

For interactions, a range of methods are available and at low tier; the HI modified by binary interactions allows hazard data for pairs of chemicals to determine the binary weight of evidence for each of these pairs, determining the expected direction of an interaction [18]. The interaction-based HI allows translation of the available interaction information into a numerical score using an algorithm based on expert judgement. The numerical score takes into account variables such as nature of interaction, data quality, toxicological plausibility of the interaction under real exposure conditions and relevance for human or animal health [34,46,47,48,49]. Limitations of this approach are discussed elsewhere and referred to as a subjective evaluation with intrinsic uncertainties [50]. As highlighted in the hazard assessment section, refinement using dosimetry to calculate risk metrics on an internal basis are suitable at high tier, such as physiologically based TK-TD modelling, internal hazard index modified by binary interactions or an internal combined margin of exposure. Many international efforts are ongoing to apply these approaches in human health, animal health and ecological area [25,42,51].

**Figure 5:**
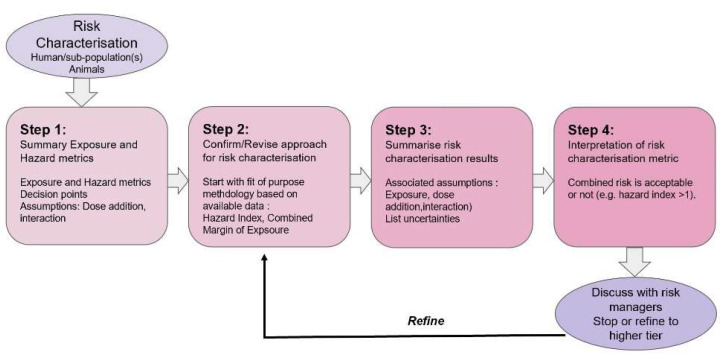
Stepwise approach for risk characterisation of combined exposure to multiple chemicals (modified from [2]).

The risk characterisation for CBA is illustrated in Figure 5 and summarised below as a step-wise approach. Note that this sequence of steps does not necessarily need to be followed in the order proposed; as indicated, the whole process is conducted in an iterative manner.

Step 1: exposure and hazard metrics from the exposure and hazard assessment are collated for each component while considering decision points from the analysis plan and assumptions, such as dose addition or interaction, for the application of the relevant methods for risk characterisation.

Step 2: an appropriate methodology for risk characterisation is applied, depending on the tier of the assessment. In this step, the approach is confirmed or not for the risk characterisation metric and interpretation. Examples of tiers and relevant methods are provided above.

Step 3: the risk characterisation results are summarised and include an overview of available data, methodologies, associated assumptions for exposure, potency, dose addition, interaction and a list of associated uncertainties.

Step 4: interpretation of the risk metric and the consideration if the level of protection is sufficient based on specific risk management considerations and procedures. When the combined risk is considered unacceptable, the risk manager will (in consultation with the risk assessor) decide to refine the assessment through higher tiers or may conclude that risk mitigation measures are required.

#### 2.3.5. Template for Summarising a Risk Assessment of Combined Exposure to Multiple Chemicals Using a Component-Based Approach

Table 1 provides a reporting table summarising consistently the results of a combined exposure to multiple chemicals using a CBA for each RA step. Such reporting should be consistent with EFSA’s general principles on transparency and reporting, including the use of the WoE approach, assessment of biological relevance, as well as the reporting and communication of uncertainties [15,35,52,53].

## 3. Scientific Criteria for Grouping Chemicals into Assessment Groups

### 3.1. Hazard-Driven Criteria for Grouping Chemicals

Hazard-driven criteria are the gold standard to group chemicals into assessment groups for the CBA. As discussed earlier, its application requires a WoE approach to assemble, weigh and integrate the available lines of evidence on chemicals’ toxicity. The recent EFSA guidance document on grouping chemicals into assessment groups [3] defines a framework to apply hazard-driven criteria for grouping chemicals into assessment groups using mechanistic information on toxicity while considering knowledge on available MoA, AOP and TK information (e.g., body burden). If such data are not available, assessment groups can then be set using data on target organ toxicity, common adverse effect or in silico predictions when no in vivo data are available [3].

Over the last decade, the grouping of chemicals has been based mainly on common MoA using available mechanistic information, as in the case of organophosphates by the US Environmental Protection Agency (US EPA) [54] and polychlorinated dibenzo-p-dioxins, dibenzofurans and dioxin-like polychlorinated biphenyls by EFSA [55]. The lowest uncertainty in grouping can be achieved when the knowledge on AOP is available, followed by MoA information for the chemicals under evaluation. In fact, chemicals sharing a common adverse outcome and for which AOPs are known should belong to the same assessment group [3].

For data-poor chemicals, the knowledge of phenomenological effects or target organ/system toxicity is often considered to define assessment groups and for grouping chemicals, thus triggering higher uncertainty. However, such chemicals can be included in an assessment group along with data-rich compounds using toxicological information derived from new approach methodologies (NAMs), such as in vitro or in silico methods. The latter can be employed to predict the effect (such as toxicity) or to group substances within a same assessment group according to common MoA information and/or structural similarity. In fact, the use of structural similarity as additional criteria for grouping of chemicals into assessment groups may reduce the overall uncertainty. In this case, to increase the confidence in the assessment of similar components, structural similarity should include more than one feature, such as chemical class, common functional groups, common precursor or breakdown products. Several software tools, such as the OECD QSAR Toolbox [56] and VEGA platform [57] (i.e., ToxWeight available within VEGA (www.vegahub.eu, accessed on 25 August 2022), can help identifying structurally related substances. Many in silico methodologies can be used for this purpose, such as molecular docking and different machine learning tools. However, the applicability domain of each model should be assessed and results from multiple models—employed for the prediction of the same property—should be integrated using WoE methods [15,58]. Overall, it is recommended to assess both similarities and dissimilarities between chemicals; this can help identifying the presence of specific chemical moieties (e.g., aromatic rings) or structural features which may impact on MoA or toxicity [3].

### 3.2. Prioritisation Methods

Prioritisation methods are used in the context of risk assessment of combined exposure to multiple chemicals to reduce the number of substances within a given assessment group. Such methods allow low-priority substances to be identified and can be particularly helpful when resources are limited and the number of chemicals high. Criteria for de-prioritisation of chemicals are the low probability of humans simultaneously exposed to certain substances from the assessment group or the marginal contribution of some substances to the combined risk. Identification of low-priority chemicals relies on a predefined cut-off value associated with an estimated contribution of each chemical to the overall risk and depends on the prioritisation method used, the availability of hazard metrics and the statistical methods applied [3,59].

Prioritisation can be performed using three main methods, depending on the context of the assessment and available data, namely a combined risk-based approach, a risk-based approach for single chemicals or an exposure-driven approach [3]. When hazard metrics for a common effect or target organ/system are accessible, then the combined risk-based approach is applied and those chemicals with a marginal contribution to the combined risk can be excluded from the grouping as low-priority chemicals. The risk-based approach for single chemicals is utilised in those cases when hazard metrics are present only for the respective critical effect of each chemical in the assessment group. Lastly, the exposure-driven approach is used to determine whether chemicals within an assessment group may co-occur and have the potential to elicit combined toxicity. It is applicable for substances without hazard metrics or when a large number of chemicals have to be evaluated. Recently, the application of a novel exposure-driven prioritisation approach has been published and illustrates its applicability for biomonitoring data focusing on multiple contaminants in human breast milk [60].

## 4. Applications in the Human Health and Animal Health Areas

### 4.1. Human Health Area

#### 4.1.1. Risk Assessment of Multiple Pesticide Residues in Food

In recent years, EFSA has conducted retrospective risk assessment of multiple pesticide residues in food, known as cumulative risk assessment, in the pesticide legislation. The methodology has been applied to pesticides causing acute and/or chronic effects on the nervous system and the thyroid using cumulative assessment groups (CAGs), which were established based on the specific effects of relevance for the combined toxicity on the target organ system as defined by EFSA [17,61]. CAGs were then refined for five specific effects on the nervous system: (i) brain and/or erythrocyte acetylcholinesterase inhibition, functional alterations of the (ii) motor, (iii) sensory, and (iv) autonomic divisions and (v) histological neuropathological changes in neural tissues [62]; and two specific effects on the thyroid: (i) hypothyroidism and (ii) parafollicular cell (C-cell) hypertrophy, hyperplasia and neoplasia [63].

The toxicological properties of all the active substances allocated in the above-mentioned CAGs were characterised for the respective specific effect through the selection of an NOAEL derived using all available information across studies, species and sexes. For each predefined CAG, an index compound was then selected to allow the calculation of relative potency factors as hazard metrics to “normalise” the toxicity of all substances within the CAG.

In parallel, exposure estimates were calculated using SAS^®^ software (SAS^®^ Enterprise Guide 7.1 and SAS^®^ Studio 3.71 (Enterprise Edition)) for different percentiles (50th, 95th, 90th and 99.9th) of the exposure distribution using monitoring data collected by EU Member States under their official 3-year monitoring programmes and individual food consumption data from 10 populations of consumer from different countries and age groups, including vulnerable ones. The threshold of regulatory consideration, as the protection goal for the CRA, was set at the 99.9th percentile by risk managers at EU level. Taking into consideration the different nature of the hazards, chronic calculations were performed for the effects on the thyroid [64], while both acute and chronic estimates were calculated for the nervous system [65,66].

For the risk characterisation, the combined margin of exposure approach was applied to each predefined CAG and derived based on exposure metrics at each percentile of the distribution and hazard metrics normalised using relative potency factors, both combined through the default assumption of dose addition, as described in the MITXOX guidance [2]. In agreement with the threshold of regulatory consideration set by risk managers at EU level, further regulatory consideration would be required when the MOET calculated at the 99.9th percentile would be below 100-fold. For each CAG, an uncertainty analysis was performed so that sources of uncertainty impacting on input data, model assumptions and assessment methodology were identified and their impact on the MOETs was quantified. The combined margin of exposure and their confidence intervals were adjusted accordingly [66,67]. In parallel, CRA were also performed and validated by the Dutch National Institute for Public Health and the Environment (RIVM) using the Monte Carlo Risk Assessment software [68,69]. Overall, based on the available data and identified uncertainties, the assessments concluded, with varying degrees of certainty, that cumulative exposures to pesticides causing effects on the nervous system and the thyroid did not reach the threshold for regulatory consideration for all the population groups considered.

Recently, a report piloted the implementation of prioritisation methods, as described above in Section 3.2, to implement in the cumulative risk assessment process as a learning tool. In essence, the cumulative risk assessment process allows the identification of chemicals contributing marginally to risk when considered individually so that they are also expected to contribute marginally to combined risk [3]. The report explored a two-step approach, namely through the identification of low-priority substances and priority target organs. First, low-priority pesticides were identified based on hazard quotient thresholds for the single substances relevant for acute effects on the nervous system or chronic effects on the thyroid using probabilistic calculations performed for 210 substances and 10 surveys. Priority pesticides were selected according to four different thresholds, namely a hazard quotient larger than 0.001, 0.01, 0.1 or 0.2 at the 99th percentile of exposure. Second, hazard metrics for each CAG were computed and risk metrics derived and compared with the risk of higher tier CAGs defined using specific effects. Overall, the approach allowed the number of pesticides to be reduced by 50% and 70% for the nervous system and thyroid CAGs, respectively, without having a substantial impact on combined margin of exposure. Overall, such prioritisation methods are expected to further lean the cumulative risk assessment process without compromising the primary goal or the exercise that is ensuring the highest possible level of protection for EU consumers [70].

#### 4.1.2. Combined Risk Assessment of Multiple Phthalates Using Biomonitoring Data

Reyes and Price [71,72] performed an assessment of human health risks from combined exposures to six phthalates, where the combined exposures were determined using biomonitoring data. The biomonitoring data used are levels of urinary metabolites of the phthalates for approximately 2500 individuals [73,74]. All data were collected over a 10-year period, in which five two-year sampling cycles were performed. The duration of this sampling programme allowed the authors to determine trends in phthalate exposures and risks over time. The sampling programme also collected data on the demographics of the surveyed individuals. These data allowed the authors to determine if any specific demographic groups were more at risk than the general population. The results of the biomonitoring are reported on the basis of each individual. While the individual’s anonymity was protected, demographic information and level of metabolites in urine were linked to a unique survey identification number. Since the metabolites of each of the six phthalates received by an individual were reported, the data supported a finding of the risk posed by combined exposures to the six phthalates for each individual. Risk characterisation was performed using two types of additive models, a relative potency factor approach [75] and the hazard index/hazard quotient approach [76].

Biomonitoring provides measures of the aggregate (total) exposures to each of the phthalates that occur as a result of exposures to multiple sources (different food items, medical devices, and consumer and industrial products). Surveying across individuals provides empirical data on variation in dose across individuals and for repeated survey data on temporal trends. Collection of demographic data on the surveyed individuals allows an investigation on the potential for the existence of sensitive subpopulations.

The following key findings from the study were highlighted by the authors:

- Interindividual variation in daily dose for the surveyed individuals varied by factors of one thousand to three thousand, depending on the phthalate.
- Children ages 6–18 had slightly larger exposures than adults on a body weight basis.
- There was no significant difference in exposure with ethnicity or gender.
- The risk predictions of the RPF and the hazard index approaches were similar.
- Only 21 of the 2663 individuals surveyed in the 2013–2014 cycle had a value of HI greater than one, suggesting that combined exposures of the six phthalates were a potential concern for less than 1% of the surveyed individuals.
- A study of the earlier cycles found that risks posed by the phthalates had declined from 2005 to 2014, largely as a result of the displacement of more toxic phthalates by less toxic phthalates.
- Only three of the six phthalates drive the hazard index values for individuals with HI values greater than one. Any future study of toxicological interactions between phthalates should focus on these phthalates.
- The differences between the largest hazard quotient, hazard index and the maximum cumulative ratio declined from 3 to 1.3 in individuals with larger HI values. This indicates that the differences between risk estimates based on response addition would be similar to those from dose addition for the individuals most at risk.

### 4.2. Animal Health Area

#### 4.2.1. Multiple Chemicals in Essential Oils

The MIXTOX guidance has also been applied to animal health RA of combined exposure to multiple chemicals in essential oils and has been illustrated within a generic case study in the guidance itself (Annex 2) and in a technical report [2,77,78]. The generic case study defined a theoretical essential oil as a mixture of botanical origin used as a flavouring feed additive in the diet of chickens for fattening (target animal species) to illustrate the methodology. Each substance in the mixture was identified and the relative amount in the essential oil determined, while co-exposure to the components of the essential oil in chickens for fattening was assumed to occur on a daily basis from hatching to 35 days. The resulting 13 substances were identified and accounted for 100% of the composition of the feed additive. A CBA was applied for the risk assessment. For exposure assessment, exposure metrics were derived on a body weight basis (mg/kg body weight per day) for each compound. The calculation combined the maximum proposed use levels of the essential oil in feed (20 mg/kg) with the maximum percentage of each chemical in the oil, corresponding, finally, with integrated feed consumption patterns in the chicken (default values: body weight (bw) 2 kg; feed intake 79 g/kg bw [79]). For hazard assessment, the grouping criteria for the multiple chemicals was based on flavouring groups and resulted in four assessment groups: flavouring groups 1, 2, 3 and 4. Reference points for each substance in each assessment group were collected from the open-source EFSA Openfood Tox Database [80], as NOAELs from sub-chronic rat studies (90 days) expressed on a body weight basis (mg/kg bw per day). When no reference points were available, read-across was applied using data for a similar chemical in the flavouring group or the 5th percentile of the distribution of the NOAELs of the corresponding Cramer Class using the threshold of toxicological concern approach. Combined toxicity was assessed using the dose addition assumption. For risk characterisation, the combined margin of exposure approach was applied using dose addition, since no evidence for interactions were available, to combine exposure metrics and reference points for each assessment group. Combined margin of exposure was interpreted as safe for the target species when above 100-fold.

From these generic case studies, the EFSA Panel on Feed and Contaminants (FEEDAP Panel) applied the approach to a number of risk assessments of multiple substances in essential oils and other preparations (e.g., extracts, oleoresins and tinctures) for use in animal species, including oregano oil [79,81], cardamom oil from *Elettaria cardamomum* (L.) Maton [82], ginger preparations [83], turmeric preparations [84], expressed lemon oil and its fractions from *Citrus limon* (L.) Osbeck and of lime oil from *Citrus aurantiifolia* (Christm.) Swingle [85], petitgrain bigarade oil from the leaves of *Citrus × aurantium* L. [86], expressed mandarin oil from the fruit peels of *C. reticulata* Blanco [87], expressed sweet orange peel oil and its fractions from *Citrus sinensis* (L.) Osbeck [88], bitter orange extract from the whole fruit of *Citrus × aurantium* L. [89], lemon extract from *Citrus limon* (L.) Osbeck [90], litsea berry oil from the fruits of *Litsea cubeba* (Lour.) Pers. [91], cinnamon tincture from the bark of *Cinnamomum verum* J. Presl [92], camphor white oil from *Cinnamomum camphora* (L.) J. Presl [93], buchu leaf oil from the leaves of *Agathosma betulina* (P.J. Bergius) Pillans [94], ylang ylang oil from the flowers of *Cananga odorata* (Lam.) Hook.f. & Thomson [95], and olibanum extract from *Boswellia serrata* Roxb. ex Colebr. [96].

#### 4.2.2. Multiple Mycotoxins in Maize

Mycotoxins, particularly as secondary metabolites of fungi, constitute prominent examples in the area of both human health and animal health, since they may cause a wide range of adverse health effects. Agricultural commodities can be contaminated with several mycotoxins produced by a plethora of fungal species, with the most relevant in food/feed safety belonging to the genera of *Aspergillus*, *Penicillium* and *Fusarium* [97]. Combined toxicity has shown these multiple mycotoxins have additive and, in some instances, even synergistic effects, a finding with significance to food safety, since 60–80% of food crops are estimated to be globally contaminated with detectable levels of mycotoxins [98]. An additional level of concern arises from the fact that precursor mycotoxins often coexist with their modified forms, with potentially unique toxicological properties, as has been demonstrated in barley, oats and wheat [99], cereal-based food and feed [100], as well as forage maize [101], among others. Concerning, EFSA established group TDI for fusarium mycotoxins, in a number of scientific opinions, to take into account combined effects of modified mycotoxins with the use of relative potency factors for zearalenone [102], deoxynivalenol [103], HT-2/T-2 toxins [104] and fumonisins [105]. Recently, animal health risk assessment of multiple mycotoxins for poultry and pigs in maize were conducted by Palumbo et al. [106], using the CBA, developed in EFSA’s MIXTOX guidance for aflatoxins, ochratoxin A, fumonisins, deoxynivalenol, zearalenone and T2/HT2 toxins [3].

Based on co-occurrence data, common source of exposure and hazard considerations, two assessment groups were set, namely, the following assessment groups were identified. With regards to ARA, the compounds were grouped by NOAEL to be conservative, since they could not be grouped by target organ or MoA due to lack of data. Exposure metrics were derived for each mycotoxin using mycotoxin occurrence data in maize for EU countries collected between 2010 and 2018 from the literature and EFSA’s database. Occurrence data were then combined for each compound with consumption data from EFSA’s comprehensive consumption database [107]. Hazard metrics, as reference points, were then collected and extracted from published EFSA Scientific Opinions structured in EFSA’s OpenFoodTox [80] from previous EFSA risk assessment (i.e., NOAEL and BMDL values). For risk characterisation, dose addition was applied and the combined margin of exposure values were derived. Two assessments were performed, namely using occurrence data from EFSA on zearalenone, deoxynivalenol and fumonisins, and deoxynivalenol and fumonisins using occurrence data from the literature. For the interpretation of combined margin of exposure with regards to deoxynivalenol and fumonisins, a value of 1 was considered as the threshold, since no uncertainty factors were needed as all reference points were available for the pig and chicken. Combined margin of exposure for poultry were greater than 1 (range: 9.6–16.5) and did not raise animal health concerns, whereas these values were below 1 in pigs (range: 0.442–0.612) and the authors concluded that animal health concern for pigs could not be excluded and in this context. Such values for zearalenone, deoxynivalenol and fumonisins assessment were above 1 in poultry (range: 25.4–68.7) and, for pigs, were close to this threshold value (range: 0.891–2.9). The authors concluded that refinement of the RA approach may be needed using, for example, an internal dose combined margin of exposure approach. RA for human health has also been performed and the reader is referred to the full manuscript for details [106].

#### 4.2.3. Multiple Pesticides in Bees

A generic case study to apply the CBA of the MIXTOX guidance to honey bee RA of combined exposure to multiple pesticides (binary mixture) has been illustrated within the guidance [2]. This case study aimed to illustrate a method to investigate deviation from the dose (concentration) addition model, such as synergistic effects. Toxic units are first derived for each chemical as a normalised value to express the relative potency of each chemical [2]. Derivation of toxic units then allowed the comparison of dose–response data from experimental toxicity studies on multiple chemicals with predictions generated using concentration addition models using the model deviation ratio. In the absence of dose–response data, the magnitude of interactions (as potency or synergism ratios) can be estimated using the estimated mean ratio as the ratio between the endpoint mean (e.g., lethal dose 50% (LD_50_)) for the single chemical and the estimated mean of the endpoint for the binary mixture [2,108]. This approach has also been applied in a meta-analysis using available data for binary mixtures in honey bees (*Apis mellifera*), wild bees (*Bombus* spp.) and solitary bee species (*Osmia* spp.). From acute mortality data on 92 binary mixtures in *Apis meliferra*, model deviation ratios showed that the concentration addition model was relevant to 17% of cases, while synergism and antagonism were observed for 72% and 11%, respectively. Since data gaps were identified, particularly for sublethal effects, a recent meta-analysis was published and proposed the use of sublethal toxicity ratios to estimate pesticide sublethal effects as the ratio between the sublethal hazard metric (low observed adverse effect level) and the LD_50_ hazard metric [109]. The author also highlighted that such datasets can be employed to develop in silico tools, such as QSAR models to predict single and multiple chemical toxicity (including sublethal effects) in bees and species of ecological relevance [110,111].

## 5. Future Challenges, Recommendations and Conclusions

RA of combined exposure to multiple chemicals is a field that has often followed an independent development pathway in various disciplines in the human health, animal health and ecological areas. This review focused on the recent EFSA guidance documents applied to this field, together with practical examples.

Many recommendations for future work have also been formulated within the guidance documents, technical reports, as well as in the recent EFSA international workshop on the topic [1,2,3,12,77,78,112]. Providing a full list of such recommendations would go beyond the scope of this review and the reader is referred to these above-mentioned documents for a comprehensive account; however, some key development and implementation needs are highlighted below. Since this special issue is focused on mycotoxins, the reader should note that most of these recommendations are applicable to a wide range of substances, including environmental contaminants and mycotoxins. The recommendations are as follows:

- Develop and maintain open-source curated databases for exposure and hazard assessment of multiple chemicals, including production, use, occurrence, consumption data, TK and toxicity in the human health, animal health and ecological areas. This will allow development, implementation and testing of the relevance of NAM-based methods, such as in silico tools in the human health and animal health area. Such open-access databases on parent compounds, metabolites associated with critical and noncritical toxicological effects, mechanistic data and TK data will support grouping, refinement of assessment groups using MoA and AOP information and the development of predictive in silico models.
- For exposure assessment: (a) develop analytical methods with a broad scope, such as nontarget chemical analysis, for simultaneously characterising concentrations of a large number of chemicals in food, feed and drinking water; (b) develop guidance for use of probabilistic methods in exposure assessment for both single and multiple chemicals; (c) develop guidance for the generation and use of biomonitoring data in exposure assessment; and (d) develop databases on human dietary and occupational exposure to multiple chemicals. In addition, recent biomonitoring programmes, such as the European research Horizon 2020 project, HBM4EU, allowed health-based guidance values to be derived from epidemiological data as human biomonitoring guidance values. It is foreseen that, in the near future, such results can also contribute to integrate biomonitoring data and epidemiological data in these databases for specific European and other world populations.
- For hazard identification and characterisation: (a) develop approaches for better integration of high throughput, in vitro and omics data generated as NAM-based datasets, as explored worldwide in translational research and Horizon 2020 programmes; (b) apply, test and implement OECD Harmonised Templates (OHT) under the OECD harmonised guidelines to support NAMs implementation and improve grouping using mechanistic data of multiple chemicals. In this context, the OHT 201 template provides means to structure intermediate effect/mechanistic data from NAM-based methods (in silico and in vitro) and integrate them in the assessment [1,2,3]; and (c) apply and implement generic physiologically based TK and TK-TD models integrating internal dose in CBAs. Examples include models developed at US-EPA and EFSA, including Httk and TKplate published on EFSA knowledge junction, respectively [113]; and (d) apply and implement biologically based models as NAMs handling TK, TD or TK-TD interactions for predicting likelihood, dose dependencies and uncertainty factors in the case of synergisms and antagonisms. A recent example is provided by the physiologically based TK-TD model investigating melamine–cyanuric acid synergism in rainbow trout [24].
- For risk characterisation: (a) testing NAM-based methods through case studies is needed and (b) the use of default threshold values for risk metrics to prioritise chemicals should be further tested depending on regulatory context, number of chemicals under consideration in the assessment and data availability.
- With regards to international scientific co-operation, further improvement between regulatory agencies, member states and international agencies is warranted through data sharing, harmonisation of methods and practice, as well as training of staff and experts.

The ecological area also requires further work, particularly to further develop and implement more holistic approaches. This has been highlighted particularly with regards to landscape modelling to integrate taxa-specific hazard information, exposure information and eco-epidemiological information in a spatial explicit fashion for different habitats and ecosystems [114]. Beyond multiple chemicals, the scientific and RA community is facing the challenge of addressing chemical stressors together with other stressors, such as temperature, nutrition and biological stressors, such as pathogens.

Recently, EFSA has addressed this issue in the bee health area through a scientific opinion illustrating a refined approach to assess combined exposure to multiple chemicals, as well as biological agents (e.g., Varroa, Nosema, deformed wing virus and acute bee paralysis virus), nutrition and beekeeping management practices, as well as environmental factors relevant to the colony (e.g., weather and floral resources). Overall, the modelling system is based on the development of ApisRAM as an agent-based simulation model allowing single chemicals, multiple chemicals and multiple stressors to be assessed at the individual and population level [115].

Research and methodological development for the RA of multiple stressors is currently undergoing development for other taxa at EFSA. Such research projects apply multidisciplinary approaches for the development of biological-based and in silico models addressing chemical toxicity and other stressors together with case studies illustrating applications in the context of RA of multiple stressors. It is foreseen that such methodologies may prove useful to address the complex challenge of multiple stressors in human health, animal health and ecological areas at the individual taxa, population, ecosystem and landscape level; still, further guidance to harmonise these approaches may be needed to ensure their implementation in regulatory RA.

Overall, the EFSA MIXTOX guidance documents provide harmonised frameworks for the RA of multiple chemicals in humans, relevant animal species, all the way to the environment through whole mixture or component-based approaches. In essence, MIXTOX brings problem formulation, exposure and hazard assessment for risk characterisation, whereas the second guidance provides hazard-driven criteria to group multiple chemicals into assessment groups. In addition, it provides prioritisation methods, including risk-based and exposure-driven methods to identify key contributors driving either risk or exposure in a given context. Applications of this guidance have been illustrated using practical RA case studies for humans and farm animal species, namely multiple pesticides and phthalates and multiple substances in essential oils, respectively. Recommendations for future work in the human and animal health area have been formulated for each step of the process using those from the MIXTOX guidance documents, as well as discussions from EFSA’s recent international workshop on the topic [1]. Finally, two major challenges beyond multiple chemicals have been identified for the environmental area. The first one reflects the need to develop more holistic approaches, allowing a move towards landscape modelling and systems-based approaches while integrating data at different levels of biological organisation (molecular, individual, species, population, ecosystem and landscape). The second major challenge requires the development of methods allowing RA of multiple stressors, integrating data for chemicals, emerging pathogens, invasive species and climate change, as demonstrated for honey bees and amphibians [115].

## Figures and Tables

**Table 1:** Summary template format for summarising a risk assessment of combined exposure to multiple chemicals using a component-based approach (modified from [2]).

**Problem Formulation**	Description of the Components in the Mixture	Chemical Space to Be Covered, Composition, Data Availability for Components
	Conceptual model	Question/Terms of Reference, Source, exposure pathways, Species/subpopulation, Regulatory framework, Other?
	Methodology	Overview of available data Component-based approach Principles for grouping and Assessment Group(s)
	Analysis plan	
**Exposure Assessment**	Components of the assessment group	
	Summary occurrence (concentration) data	
	Summary exposure	Assumptions, Exposure metrics
		Identify uncertainties
**Hazard Identification and Hazard** **Characterisation**	Component-based approach	
	Reference points/Reference values	
	Summary hazard metrics	Assumptions combined toxicity (Dose addition, response addition, interactions) Hazard metrics
		Identify uncertainties
**Risk** **Characterisation**	Summary exposure and hazard metrics	
	Risk characterisation approach	
	Summary risk metrics	Associated Assumptions (Dose addition, response addition, interactions), Risk metrics
		Overall uncertainty analysis
	Interpretation	

## Data Availability

Not applicable.
